# Trends in hospitalizations and emergency department visits among women with hyperglycemia in pregnancy between 2008 and 2017 in Taiwan

**DOI:** 10.3389/fendo.2022.1005722

**Published:** 2022-11-24

**Authors:** Jun-Sing Wang, Ming-Chu Chin, Jung-Fu Chen, Chien-Ning Huang, Chii-Min Hwu, Horng-Yih Ou, Yi-Sun Yang, Chih-Cheng Hsu, Chih-Yuan Wang

**Affiliations:** ^1^ Division of Endocrinology and Metabolism, Department of Internal Medicine, Taichung Veterans General Hospital, Taichung, Taiwan; ^2^ Department of Medicine, School of Medicine, National Yang Ming Chiao Tung University, Taipei, Taiwan; ^3^ Department of Post-Baccalaureate Medicine, College of Medicine, National Chung Hsing University, Taichung, Taiwan; ^4^ Institute of Biomedical Sciences, National Chung Hsing University, Taichung, Taiwan; ^5^ Institute of Population Health Sciences, National Health Research Institutes, Miaoli, Taiwan; ^6^ Division of Endocrinology and Metabolism, Department of Internal Medicine, Kaohsiung Chang Gung Memorial Hospital, Kaohsiung, Taiwan; ^7^ School of Medicine, College of Medicine, Chang Gung University, Taoyuan, Taiwan; ^8^ Institute of Medicine, School of Medicine, Chung Shan Medical University, Taichung, Taiwan; ^9^ Section of Endocrinology and Metabolism, Department of Medicine, Taipei Veterans General Hospital, Taipei, Taiwan; ^10^ Division of Endocrinology and Metabolism, Department of Internal Medicine, National Cheng Kung University Hospital, Tainan, Taiwan; ^11^ College of Medicine, National Cheng Kung University, Tainan, Taiwan; ^12^ Division of Endocrinology and Metabolism, Department of Internal Medicine, Chung Shan Medical University Hospital, Taichung, Taiwan; ^13^ National Center for Geriatrics and Welfare Research, National Health Research Institutes, Miaoli, Taiwan; ^14^ Department of Health Services Administration, China Medical University, Taichung, Taiwan; ^15^ Department of Family Medicine, Min-Sheng General Hospital, Taoyuan, Taiwan; ^16^ Department of Internal Medicine, National Taiwan University Hospital, Taipei, Taiwan

**Keywords:** diabetes mellitus, gestational diabetes mellitus, hospitalization, hyperglycemia in pregnancy, emergency department

## Abstract

**Introduction:**

We investigated health service utilization, including hospitalizations and emergency department visits, for women with hyperglycemia in pregnancy between 2008 and 2017 in Taiwan.

**Methods:**

Data from the Health and Welfare Data Science Center were used to conduct this nationwide population-based study. We identified pregnant women and the date of childbirth according to Birth Certificate Applications from 2007 to 2018. The study population was divided into four groups: known DM, newly diagnosed DM, GDM, and no DM/GDM. To assess quality of healthcare during the gestation period, trends in 30-day readmission rate, number of emergency department visits/hospitalizations per 100 childbirths, and length of hospital stay from 2008 to 2017 were examined.

**Results:**

A total of 1830511 childbirths and 990569 hospitalizations were identified for analyses. Between 2008 and 2017, women with hyperglycemia in pregnancy (known DM, newly diagnosed DM, and GDM) had a higher rate of hospitalization, a longer length of hospital stay, and higher rates of various maternal and fetal outcomes, compared with women with no DM/GDM. Nevertheless, the differences between women with GDM and those with no DM/GDM in the aforementioned outcome measures were modest. Women with GDM had a modest decrease in the 30-day readmission rate (p for trend 0.046) with no significant difference in the number of emergency department visits during the study period.

**Discussion:**

Our findings provide evidence of the quality of healthcare for women with GDM between 2008 and 2017 in Taiwan.

## Introduction

Diabetes mellitus (DM) has been associated with adverse pregnancy outcomes (1, 2). Even in women with no history of DM, hyperglycemia may be noted during the gestation period, especially in those with risk factors (3). The diagnosis of gestational diabetes mellitus (GDM) is usually confirmed using an oral glucose tolerance test between 24 weeks and 28 weeks of gestation (3, 4). Similar to pregnant women with preexisting DM, women with GDM have a higher risk of adverse maternal and neonatal outcomes (5–7). Furthermore, glycemic control for GDM may improve outcomes (8, 9). Hence, screening for GDM in women at risk is recommended (3, 10).

Given the various screening tests used in clinical practice (4, 11–14), the prevalence of GDM varies widely in previous studies (15, 16). According to a recent report (17) from the International Diabetes Federation, around 21.1 million (16.7%) of live births in 2021 were to women who had hyperglycemia during the pregnancy. Among these, most (80.3%) were due to GDM. Moreover, more than half of the affected live births were in the South-East Asia and Western Pacific regions (17). In a recent study (18), the prevalence of GDM in Taiwan has increased from 7.6% in 2004 to 13.4% in 2015. Therefore, improving healthcare for women with GDM has emerged as an important issue (19).

Despite the rapid increase in the prevalence of GDM, data on healthcare resource utilization by women with GDM are scarce. Most of the healthcare costs for women with diabetes during pregnancy were attributed to hospital inpatient care, according to a recent study (20). In this study, we aimed to investigate health service use, including hospitalizations and emergency department visits, for women with hyperglycemia during pregnancy in Taiwan.

## Materials and methods

### Study ethics and database

Data from the Health and Welfare Data Science Center were used to conduct this population-based study. We identified pregnant women and the date of childbirth according to Birth Certificate Applications from 2007 to 2018. Relevant data (including diagnosis, treatment, outpatient clinic visits, emergency department visits, and hospitalizations) were retrieved by linking to the Registry for Beneficiaries, Ambulatory Care Expenditures by Visits, and Inpatient Expenditures by Admissions. This study was approved by the Research Ethics Committee of the National Health Research Institutes (EC1110505-E), and the requirement for patient consent was waived as the retrospective data were de-identified prior to analyses.

### Diagnostic categories of hyperglycemia in pregnancy

The date of childbirth was defined as the end of the gestation period. The study population was divided into four groups according to the diagnosis of diabetes at an outpatient clinic visit or in a hospitalization:

1. Known DM: a diagnosis of DM (ICD-9-CM Codes 250 or ICD-10-CM Codes E08-E13) was noted within one year prior to the start of the gestation period.
2. Newly diagnosed DM: a diagnosis of DM (ICD-9-CM Codes 250 or ICD-10-CM Codes E08-E13, O24.0, O24.1, O24.3, O24.8) was noted between the start and the 24^th^ week of the gestation period.
3. GDM: a diagnosis of DM (ICD-9-CM Codes 250, 648.00-648.04, or ICD-10-CM Codes E08-E13, O24.4, O24.9, O99.810, O99.814, O99.815) was noted between the 24^th^ week and the end of the gestation period.
4. No DM/GDM: no DM (any of the above diagnosis codes) was noted between one year prior to the start of the gestation period and the date of childbirth.

The Diabetes Association of the Republic of China (DAROC) (http://www.endo-dm.org.tw/dia/) recommends universal screening for all pregnant women with no history of DM. Screening for newly diagnosed DM is suggested using fasting plasma glucose (≥ 126 mg/dl) or glycated hemoglobin (HbA1c) (≥ 6.5%) before gestational week 24. Screening for GDM is suggested using either one-step or two-step approach at gestational week 24-28. For one-step approach, a 75-g oral glucose tolerance test is administered and plasma glucose is measured at three time points (fasting, 1 hour, and 2 hour). The thresholds of plasma glucose for each time point are 92, 180, and 153 mg/dl, respectively (21). GDM is diagnosed if any one of the plasma glucose is higher than the threshold. For two-step approach, a 50-g glucose challenge test is conducted. Plasma glucose is measured at 1 hour, and the threshold is 130 (90% sensitivity) or 140 (80% sensitivity) mg/dl. A 100-g oral glucose tolerance test is conducted for women who have a positive 50-g glucose challenge test. Plasma glucose is measured at four time points (fasting, 1 hour, 2 hour, and 3 hour). The thresholds of plasma glucose for each time point are 95, 180, 155, and 140 mg/dl, respectively (22). GDM is diagnosed if any two of the plasma glucose is higher than the threshold. Medical care for women with hyperglycemia in pregnancy is usually provided by an obstetrician and an endocrinologist. The therapeutic targets for these women are HbA1c < 6.0-7.0% (if this can be achieved without significant hypoglycemia), fasting glucose ≤ 95 mg/dl, 1-hour postprandial glucose ≤ 140 mg/dl, and 2-hour postprandial glucose ≤ 120 mg/dl (23).

### Outcomes of interest

Health service utilization, including hospitalizations and emergency department visits, within the gestation period was analyzed. We defined the first discharge diagnosis code as the primary diagnosis, and the causes of hospitalization were grouped as obstetric-related (ICD-9-CM Codes 630-639, 640-646, 651-676, 760-779, or ICD-10-CM Codes O00-O99, P00-P96), diabetes-related (ICD-9-CM Codes 250.1-250.3, 250.8, 251.0-251.2, or ICD-10-CM Codes E08.0, E09.0, E10.1, E10.62-E10.65, E10.69, E11.0, E11.6, E13.0, E13.11, E08.641, E09.641, E10.610, E10.618, E13.641), and others. Causes of emergency department visits were also grouped as obstetric-related (ICD-9-CM Codes 630-639, 640-647, 650-669, or ICD-10-CM Codes N96, O00-O04, O07-O09, O10-O16, O20, O21, O23, O26, O30-O36, O40-O48, O60-O66, O69, O70-O76, O80, O89, O98, O99.21, Z33), diabetes-related (ICD-9-CM Codes 250 or ICD-10-CM Codes E08, E11, E13), and others. Among diabetes-related causes, hyperosmolar hyperglycemic state (HHS)/diabetic ketoacidosis (DKA) (ICD-9-CM Codes 250.10, 250.12, 250.20, 250.22 or ICD-10-CM Codes E11.00, E11.01, E11.65, E11.69) and hypoglycemia (ICD-9-CM Codes 250.30, 250.32, 251.0, 251.1, 251.2 or ICD-10-CM Codes E16.0-E16.2, E11.641) were identified and reported.

To assess quality of healthcare during the gestation period, we examined the 30-day readmission rate, number of emergency department visits/hospitalizations per 100 childbirths, and length of hospital stay. The 30-day readmission rate (%) was calculated as the number of 30-day readmissions*100 divided by the number of hospitalizations. The number of emergency department visits (or hospitalizations) per 100 childbirths was calculated as the number of emergency department visits (or hospitalizations)*100 divided by the number of childbirths. Maternal (pregnancy induced hypertension, pre-eclampsia, and prolonged labor) and fetal (preterm birth [before 37 weeks of pregnancy], birth weight <10^th^ percentile, birth weight >90^th^ percentile, and hypoglycemia) outcomes were determined and compared among the four groups.

### Statistical analysis

Data manipulation and statistical analyses were performed using SAS version 9.4 (SAS Institute, Cary, NC, USA). Data were examined according to the diagnostic categories (known DM, newly diagnosed DM, GDM, and no DM/GDM) and maternal age (<30 years, 30-<35 years, and ≥ 35 years). Statistical differences in the number of hospitalizations per 100 childbirths, length of hospital stay, and causes of hospitalizations across the diagnostic categories were examined using one-way ANOVA. Trends of 30-day readmission rate and number of emergency department visits per 100 childbirths from 2008 to 2017 were examined for statistical significance (p for trend). A p value of less than 0.05 was considered statistical significance.

## Results

A total of 1830511 childbirths and 990569 hospitalizations were identified for analyses. **Table 1** shows the number of childbirths, hospitalizations during gestation period, and length of hospital stay from 2008 to 2017. The total number of childbirths in each year was relatively stable during the study period. However, the proportion of maternal age <30 years declined from 47.07% in 2008 to 30.97% in 2017. Conversely, the rate of maternal age ≥ 35 years increased from 15.78% to 32.13%. Similar findings were noted regarding the total number and maternal age distribution of hospitalizations during the gestation period. The number of hospitalizations per 100 childbirths was around 53-55, and the mean length of hospital stay was 4-5 days.

**Table 1:** Number of childbirths, hospitalizations, and length of hospital stay during gestation period from 2008 to 2017.

	2008	2009	2010	2011	2012	2013	2014	2015	2016	2017
Number of childbirths	179881	175302	151306	179607	211768	176776	192206	195612	189580	178473
Maternal age <30 years	47.07%	43.44%	39.51%	36.78%	34.90%	32.71%	31.45%	30.90%	30.89%	30.97%
Maternal age 30 to <35 years	37.15%	39.63%	40.37%	42.05%	43.19%	41.86%	42.17%	40.96%	38.68%	36.90%
Maternal age ≥ 35 years	15.78%	16.92%	20.12%	21.17%	21.91%	25.43%	26.38%	28.15%	30.42%	32.13%
Number of hospitalizations	96108	94302	84084	98293	113872	96034	103869	106543	99754	97710
Maternal age <30 years	46.0%	42.6%	38.8%	35.9%	34.1%	32.0%	30.7%	30.4%	30.4%	30.7%
Maternal age 30 to <35 years	37.6%	39.8%	40.3%	42.1%	43.1%	41.6%	42.2%	40.6%	38.2%	36.1%
Maternal age ≥ 35 years	16.4%	17.6%	20.9%	22.0%	22.7%	26.4%	27.1%	29.0%	31.4%	33.2%
Number of hospitalizations per 100 childbirths	53.4	53.8	55.6	54.7	53.8	54.3	54.0	54.5	52.6	54.7
Known DM	66.3	70.4	72.2	69.4	65.3	64.4	67.2	67.2	62.9	68.6
Newly diagnosed DM	70.3	85.8	80.1	87.4	74.4	85.7	76.8	69.4	67.1	74.0
GDM	56.6	56.6	59.2	57.6	56.2	56.8	56.1	56.9	55.2	57.0
No DM/GDM	52.9	53.2	54.8	54.1	53.2	53.7	53.5	53.8	52.0	54.1
Length of hospital stay, days	4.69	4.77	4.91	4.92	4.79	4.89	4.90	4.84	4.18	4.87
Known DM	6.41	6.56	5.84	6.82	6.87	6.19	7.03	6.89	4.97	6.89
Newly diagnosed DM	5.56	6.85	7.26	6.67	6.21	6.78	7.05	5.54	4.56	5.82
GDM	4.78	4.90	4.99	4.96	4.87	4.91	4.88	4.87	4.23	4.92
No DM/GDM	4.63	4.69	4.83	4.87	4.70	4.84	4.85	4.78	4.14	4.83

Data are presented as number or %. DM, diabetes mellitus; GDM, gestational diabetes mellitus.

**Table 2** shows the number of hospitalizations per 100 childbirths, length of hospital stay, and the most frequent causes of hospitalizations between 2008 and 2017, according to the diagnostic categories. Overall, the number of hospitalizations per 100 childbirths was higher in women with known DM (67.16), newly diagnosed DM (75.90), and GDM (56.74), compared with no DM/GDM (53.49). The findings were similar across the maternal age subgroups. The length of hospital stay was longer in women with known DM (6.47 ± 8.92), newly diagnosed DM (6.03 ± 8.66), and GDM (4.84 ± 5.42), compared with no DM/GDM (4.71 ± 6.32). The main causes of hospitalizations were obstetric-related (~70%), while few hospitalizations were diabetes-related (<1%).

**Table 2:** Number of hospitalizations, length of hospital stay, and causes of hospitalization during the gestation period, 2008-2017.

	Known DM	Newly diagnosed DM	GDM	No DM/GDM
Maternal age, years	32.97 ± 4.95*	32.81 ± 5.04*	32.29 ± 4.69*	30.94 ± 4.89
Number of hospitalizations per 100 childbirths	67.16*	75.90*	56.74*	53.49
Maternal age <30 years	61.57*	64.22	51.60*	48.79
Maternal age 30 to <35 years	59.70*	65.12*	51.78*	49.43
Maternal age ≥ 35 years	61.08*	66.41*	52.14*	48.98
Length of hospital stay, days	6.47 ± 8.92*	6.03 ± 8.66*	4.84 ± 5.42*	4.72 ± 6.32
Maternal age <30 years	5.79 ± 6.62*	5.55 ± 7.30*	4.40 ± 4.49*	4.52 ± 5.50
Maternal age 30 to <35 years	6.35 ± 8.59*	5.89 ± 7.88*	4.77 ± 5.35*	5.03 ± 6.94
Maternal age ≥ 35 years	7.00 ± 10.28*	6.49 ± 10.12*	5.36 ± 7.74*	5.68 ± 8.91
Causes of hospitalization, %				
Obstetric related	71.90%	71.76%	69.81%	67.55%
Diabetes related	0.66%	0.52%	0.01%	—
Others				
Renal disease	0.54%	0.64%	0.19%*	0.22%
Respiratory disease	0.32%*	0.67%	0.18%*	0.20%
Pneumonia	0.28%	0.26%*	0.06%*	0.07%

Data are presented as mean ± SD, number, or %. DM, diabetes mellitus; GDM, gestational diabetes mellitus. *p < 0.05 vs. No DM/GDM.

**Figure 1:**
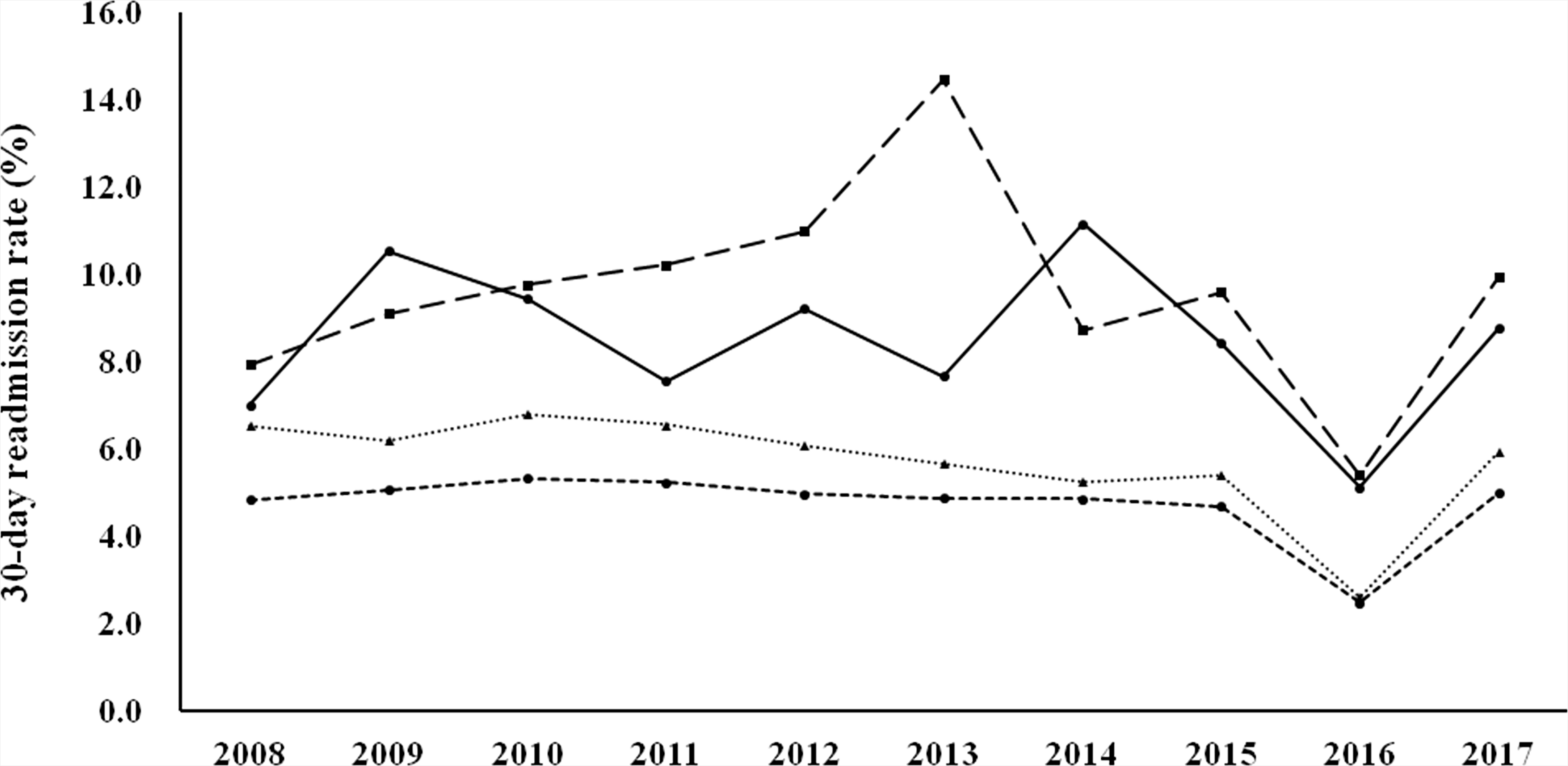
30-day readmission rates from 2008 to 2017 according to the diagnostic categories. Circle spots and solid line, known DM. Square spots and dashed line, newly diagnosed DM. Triangle spots and dashed line, GDM. Circle spots and dashed line, no DM/GDM. P for trend 0.046 for GDM.

**Figure 2:**
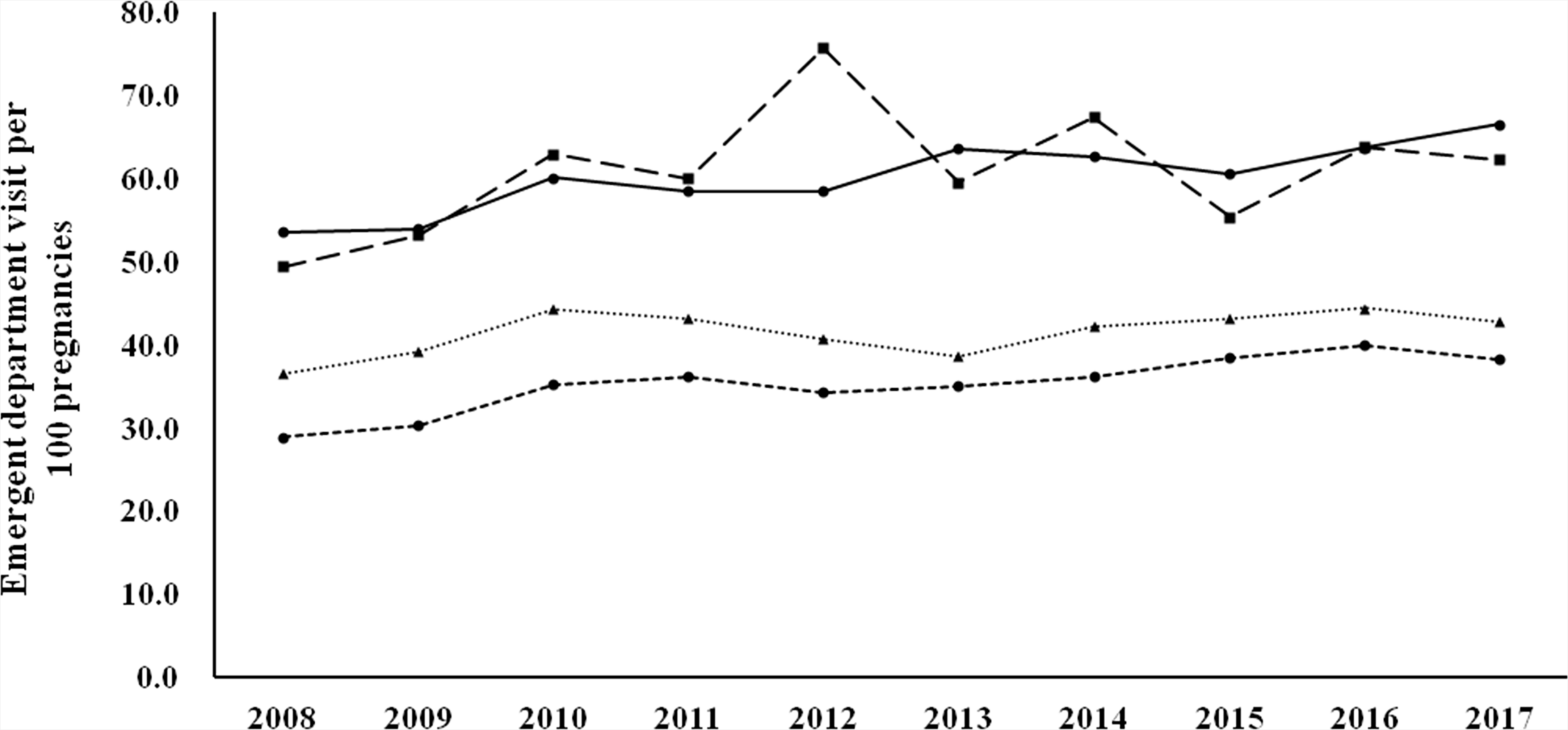
Number of emergency department visits per 100 childbirths from 2008 to 2017 according to the diagnostic categories. Circle spots and solid line, known DM. Square spots and dashed line, newly diagnosed DM. Triangle spots and dashed line, GDM. Circle spots and dashed line, no DM/GDM. P for trend <0.001 for known DM and no DM/GDM.

**Figure 1** shows the 30-day readmission rates according to the diagnostic categories. Women with known DM and newly diagnosed DM had a higher 30-day readmission rate than those with GDM and no DM/GDM. The trends were similar and the changes were modest during the study period (there was a modest decrease in women with GDM, p for trend 0.046). **Figure 2** shows the number of emergency department visits per 100 childbirths according to the diagnostic categories. Women with known DM and newly diagnosed DM were more likely to have an emergency department visit than those with GDM and no DM/GDM. The number significantly increased from 2008 to 2017 in women with known DM and no DM/GDM (both p for trend <0.001), but not in those with newly diagnosed DM and GDM.

**Table 3** shows the most frequent causes of emergency department visits according to the diagnostic categories. Causes other than obstetric-related and diabetes-related constituted 60-65% of the emergency department visits. Among these, the most frequent cause was unspecified abdominal pain. Around 35% were obstetric-related, among which the most frequent cause was hemorrhage in early pregnancy, followed by early or threatened labor. Among the diabetes-related emergency department visits, the rate of hypoglycemia was higher than that of acute hyperglycemia (HHS or DKA).

**Table 3:** Most frequent causes for emergency department visits during gestation period, 2008-2017.

	Known DM	Newly diagnosed DM	GDM	No DM/GDM
Obstetric related, %	33.67	35.23	39.16	33.89
Diabetes related, %	5.16	1.29	0.14	—
Others, %	61.17	63.48	60.70	66.10
Obstetric related, %				
Hemorrhage in early pregnancy	37.03	35.07	35.07	32.62
Early or threatened labor	28.29	26.98	26.98	28.85
False labor	2.41	1.44	1.44	2.10
Other complications of pregnancy	4.08	1.35	1.35	4.41
Diabetes related, %				
HHS or DKA	4.37	3.03	2.04	—
Hypoglycemia	23.91	3.03	6.80	—
Others	71.72	93.94	91.16	—
Others, %				
Unspecified abdominal pain	15.01	17.13	17.27	16.16
Noninfective gastroenteritis and colitis	4.99	5.03	5.94	5.85
Fever	4.56	4.78	4.73	4.74

Data are presented as %. DKA, diabetic ketoacidosis; DM, diabetes mellitus; GDM, gestational diabetes mellitus; HHS, hyperosmolar hyperglycemic state.

Maternal and fetal outcomes among the four groups are shown in **Table 4**. There were significant differences between women with hyperglycemia in pregnancy (known DM, newly diagnosed DM, and GDM) and those with no DM/GDM in these outcomes. Nevertheless, the differences between women with GDM and those with no DM/GDM were modest. For example, the rates of preterm birth (before 37 weeks of pregnancy) were 23.18%, 20.01%, 9.92%, and 9.76%, respectively.

**Table 4:** Maternal and fetal outcomes by diagnosis of diabetes, 2008-2017.

	Known DM	Newly diagnosed DM	GDM	No DM/GDM
Maternal outcomes, per 100 childbirths				
Pregnancy induced hypertension	28.06*	27.98*	5.31*	2.54
Pre-eclampsia	8.58*	8.86*	3.62*	1.85
Prolonged labor	5.08*	5.87*	4.52*	4.22
Fetal outcomes, %				
Preterm birth (before 37 weeks of pregnancy)	23.18*	20.01*	9.92*	9.76
Birth weight <10^th^ percentile	9.17*	9.85	9.31*	10.59
Birth weight >90^th^ percentile	23.53*	19.62*	10.02*	6.82
Hypoglycemia	3.47*	2.02*	1.29*	1.09

Data are presented as number or %. DM, diabetes mellitus; GDM, gestational diabetes mellitus. *p < 0.05 vs. No DM/GDM.

## Discussion

In this study, we examined health service utilization in women with various glucose regulation states (known DM, newly diagnosed DM, GDM, and no DM/GDM) during the gestation period from 2008 to 2017. We found that the rate of maternal age ≥ 35 years progressively increased during the study period (from 15.78% in 2008 to 32.13% in 2017, **Table 1**). Women with hyperglycemia in pregnancy (known DM, newly diagnosed DM, and GDM) had a higher rate of hospitalization, a longer length of hospital stay, and higher rates of various maternal and fetal outcomes, compared with women with no DM/GDM (**Tables 2**, **4**). Nevertheless, the differences between women with GDM and those with no DM/GDM in the aforementioned outcome measures were modest. Furthermore, women with GDM had a modest decrease in the 30-day readmission rate (p for trend 0.046, **Figure 1**), with no significant difference in the number of emergency department visits (**Figure 2**) between 2008 and 2017. Our findings provide evidence of the quality of healthcare for women with GDM in Taiwan.

Preexisting DM and GDM in women have been associated with adverse maternal and neonatal outcomes (1, 24–26). However, there are few data on health service utilization with respect to hospitalizations and emergency department visits in women with known DM and GDM during the gestation period. In a recent report (20), women with diabetes had a higher healthcare expenditure during pregnancy than those without diabetes, and most of the expenditure was attributed to hospital inpatient cost. Our findings showing that women with known DM and newly diagnosed DM had a higher number of hospitalizations and a longer length of hospital stay than women with no DM/GDM during the gestation period (**Table 2**) are in line with the aforementioned results (20). The differences were modest (number of hospitalizations per 100 childbirths 56.74 vs. 53.49, length of hospital stay 4.84 ± 5.42 vs. 4.72 ± 6.32) between women with GDM and those with no DM/GDM. Similar findings were noted regarding the maternal and fetal outcomes among the four groups (**Table 4**). Since the prevalence of GDM significantly increased from 2004 (7.6%) to 2015 (13.4%) in Taiwan (18), our findings provide an important reference for healthcare quality in women with GDM.

It is interesting to note that the number of emergency department visits per 100 childbirths increased from 2008 to 2017 (p for trend <0.001 for known DM and no DM/GDM), while there was no significant increase in 30-day readmission rates (a modest decrease in GDM group, p for trend 0.046) during the same period. The increase in emergency department utilization in pregnant women merits further investigation. We found that less than 40% of the emergency department visits were due to obstetric or diabetes-related causes (**Table 3**). In contrast, most of the hospitalizations (~70%) during the same period were due to obstetric-related causes (**Table 2**). It seems that hospitalization was usually not required for emergency department visits that were unrelated to obstetric-causes. With regard to diabetes-related emergency department visits, the rate of hypoglycemia was higher than acute hyperglycemia (HHS or DKA) (**Table 3**). As insulin therapy is recommended as the first-line pharmacologic treatment for preexisting diabetes (27) and GDM (4), our findings suggest that interventions to decrease emergency department utilization due to hypoglycemia should be considered to improve healthcare quality for women with hyperglycemia during pregnancy.

The main strengths of this study were the use of a nationwide, population-based dataset and a large study population. Nevertheless, there were several limitations in this study. First, we did not have data on maternal body mass index. Second, laboratory data related to glycemic control (such as fasting plasma glucose or HbA1c) were not available. Third, some confounding factors, such as nulliparous, urbanization of residence city (which may affect healthcare accessibility), primary or tertiary care, and socioeconomic status were not addressed. All of the aforementioned factors (28–30) may influence healthcare resource utilization during the gestation period. Final, we cannot be sure if universal screening for GDM was carried out in all medical institution since our analyses were based on retrospectively collected data. The prevalence of GDM in Taiwan increased from 7.6% in 2004 to 13.4% in 2015 (18). In a recent pragmatic, randomized clinical trial of GDM screening (31), the prevalence of GDM was 16.5% based on one-step approach (maternal age 29.4 ± 5.5 years) and 8.5% based on two-step approach (maternal age 29.3 ± 5.5 years). Thus we suggest that the screening for GDM in Taiwan should have been conducted in an efficient manner to disclose an increase in the prevalence of GDM. Despite these limitations, our results based on 10-year nationwide data still have important implications for the medical care of women with hyperglycemia in pregnancy.

In conclusion, the rate of maternal age ≥ 35 years significantly increased from 2008 (15.78%) to 2017 (32.13%) in Taiwan. Among women with GDM, there was a modest increase in the number of hospitalizations per 100 childbirths (56.74 vs. 53.49) and length of hospital stay (4.84 ± 5.42 vs. 4.72 ± 6.32) compared with those with no DM/GDM. From 2008 to 2017, women with GDM had a modest decrease in the 30-day readmission rate (p for trend 0.046) with no significant difference in the number of emergency department visits. Our findings provide evidence of healthcare quality for women with GDM in Taiwan.

## Data availability statement

The datasets presented in this article are not readily available because of privacy/ethical restrictions. Requests to access the datasets should be directed to Chih-Cheng Hsu, cch@nhri.edu.tw.

## Ethics statement

The studies involving human participants were reviewed and approved by the Research Ethics Committee of the National Health Research Institutes. Written informed consent for participation was not required for this study in accordance with the national legislation and the institutional requirements.

## Author contributions

J-SW, J-FC, C-NH, C-MH, and C-YW contributed to conception and design of the study. M-CC, H-YO, Y-SY, and C-CH organized the database. M-CC and C-CH performed the statistical analysis. J-SW, M-CC, and C-CH wrote the first draft of the manuscript. J-FC, C-NH, C-MH, H-YO, Y-SY, and C-YW reviewed and edited the manuscript. All authors contributed to the article and approved the submitted version.

## Funding

This research was funded by the Diabetes Association of the Republic of China [grant number DAROC2021ATLAS-0001, 2021] and the Taiwanese Association of Diabetes Educators [grant number TADE-2021-RES-01, 2021]. The sponsors had no role in the design, execution, interpretation, or writing of the study.

## Acknowledgments

We thank the Health and Welfare Data Science Center for providing data and Institute of Population Health Sciences, National Health Research Institutes for performing analysis.

## Conflict of interest

The authors declare that the research was conducted in the absence of any commercial or financial relationships that could be construed as a potential conflict of interest.

## Publisher’s note

All claims expressed in this article are solely those of the authors and do not necessarily represent those of their affiliated organizations, or those of the publisher, the editors and the reviewers. Any product that may be evaluated in this article, or claim that may be made by its manufacturer, is not guaranteed or endorsed by the publisher.
